# The clinical challenge of MEN1 phenocopies: insights from a multicentric national retrospective study

**DOI:** 10.1007/s40618-025-02743-w

**Published:** 2025-11-12

**Authors:** Rosaria M. Ruggeri, Elio Benevento, Iderina Hasballa, Erika Maria Grossrubatscher, Roberta Modica, Manuela Albertelli, Bianca Golisano, Vito Guarnieri, Flavia Pugliese, Valentina Guarnotta, Simona Jaafar, Andrea Lania, Antonio Prinzi, Isabella Zanata, Maria Chiara Zatelli, Annamaria Colao, Antongiulio Faggiano

**Affiliations:** 1 Department of Human Pathology DETEV, Unit of Endocrinology, University of Messina, Messina, Italy; 2 Endocrinology, Diabetology and Andrology Unit, Department of Clinical Medicine and Surgery, Federico II University of Naples, Naples, Italy; 3 Endocrinology Unit, Department of Internal Medicine and Medical Specialties (DIMI), University of Genova, Genova, Italy; 4 Endocrine Unit, ASST Grande Ospedale Metropolitano Niguarda, Milano, Italy; 5 Endocrinology Unit, Department of Clinical and Molecular Medicine, ENETS Center of Excellence, Sant’Andrea University Hospital, Sapienza University of Rome, Rome, Italy; 6 Division of Medical Genetics, Fondazione IRCCS Casa Sollievo della Sofferenza, San Giovanni Rotondo, Italy; 7 Unit of Endocrinology, Department of Health Promotion, Mother and Child Care, Internal Medicine and Medical Specialties, Policlinico Paolo Giaccone, Università degli studi di Palermo, Palermo, Italy; 8 Unit of Endocrinology, IRCCS Humanitas Research Hospital, Rozzano, Milan Italy; 9 Endocrinology Unit, Department of Clinical and Experimental Medicine, Medical Center, University of Catania, Catania, Garibaldi-Nesima, Italy; 10 Department of Precision Medicine in Medical, Surgical and Critical Care (Me.Pre.C.C.), University of Palermo, Palermo, Italy; 11 Section of Endocrinology, Geriatrics and Internal Medicine, Department of Medical Sciences, University of Ferrara, Ferrara, Italy

**Keywords:** Multiple endocrine neoplasia type 1 (MEN1), Phenocopy, Primary hyperparathyroidism, Pituitary adenomas, Neuroendocrine tumors, Genetic analysis

## Abstract

Multiple endocrine neoplasia type 1 (MEN1) is an autosomal dominant disorder caused by *MEN1* gene mutations, typically involving primary hyperparathyroidism (PHPT), pancreatic neuroendocrine tumors (PanNETs), and/or pituitary neuroendocrine tumors (PitNETs). However, 10–30% of patients with MEN1-like features lack identifiable *MEN1* mutations and are classified as phenocopies. This retrospective multicenter study, conducted across 10 Italian referral centers, aimed to characterize the main clinical features of phenocopies. Among 240 patients evaluated for suspected MEN1 over five years, 175 (mean age 43.2 ± 19.7; 101 females) had genetically confirmed MEN1, while 65 (27%; mean age 59.9 ± 11.6; 44 females) were identified as phenocopies. Of these, 46 (70.7%) were also negative for *CDKN1B* mutations, confirming the rarity of MEN4. Phenocopies were diagnosed one to two decades later than MEN1 patients (*p* < 0.0001). PHPT was the most frequent manifestation in both groups (80% of phenocopies vs. 81% of MEN1), but tumor associations differed significantly between groups (*p* < 0.001): 41% of MEN1 patients showed the classic triad, compared to only 1% of phenocopies; PHPT with NETs was more common in MEN1 (32%), whereas PHPT with PitNETs occurred more often in phenocopies (54%), reflecting patterns of sporadic tumors. Notably, 11% of phenocopies had a first-degree relative with MEN1-related diseases, and 51% had a personal or family history of cancer. In conclusion, MEN1 phenocopies are relatively common and represent a clinical challenge. Given their distinct features and familial backgrounds, an extended genetic panel should be offered to these patients together with periodical screening of MEN1-related disease.

## Introduction

Multiple endocrine neoplasia type 1 (MEN1) is an autosomal dominant hereditary disorder with an estimated prevalence of 1:30,000 individuals [1]. It represents the most common MEN syndrome [2]. MEN1 is caused by germline loss-of-function mutations in the tumor-suppressor gene *MEN1*, localized on chromosome 11q13, which encodes the menin protein, involved in cell cycle regulation. Mutations could occur in any site of the nine coding exons, and nowadays more than 1,800 *MEN1* gene pathogenetic variants have been reported [3]. Penetrance of MEN1 is high: over 90% of individuals carrying a *MEN1* mutation will present clinical manifestations [1]. Clinically, the syndrome is characterized by the combined occurrence of 2 or more of the following classical endocrine tumors: (a) parathyroid hyperplasia/adenomatosis with primary hyperparathyroidism (PHPT, >95%), (b) pancreatic neuroendocrine tumors (PanNET, about 80%), and (c) pituitary NET (PitNET, about 30–50%), but varying combinations of more than 20 endocrine and non-endocrine tumors have been reported [1, 4]. Moreover, the syndrome is characterized by a high inter- and intra-familial variability in clinical presentation, with no established correlation genotype-phenotype, and the involvement of different glands may be asynchronous [1]. As a consequence, a delay in identifying both the index case and affected family members can occur. A recent review of the Italian MEN1 registry revealed that the average age of the first MEN1 manifestation was 41.6 years, while the average age of MEN1 diagnosis was 55.1 years, with a lag time between first manifestation and diagnosis of MEN1 of more than 10 years [5]. The main challenges that can make difficult a timely diagnosis and treatment of MEN1, with potential harm to the patient, are the lack of a genotype-phenotype correlation, and the existence of phenocopies.

Phenocopy is defined as a condition in which clinical phenotype resembles a genetic disorder in an individual who is not a carrier of that specific genotype. Noteworthy, 10 to 30% of patients with MEN1-like phenotypes (clinical MEN1) do not have *MEN1* pathogenic mutations or deletions [4]. The use of second-level highly sensitive genetic tests like multiplex ligation-dependent probe amplification (MLPA) and the next generation sequencing (NGS) expanded panel, reduce the genetic negativity to a 10% of clinical MEN1 [1]. Mutations in the tumor-suppressor gene *CDKN1B* gene, encoding the cell cycle inhibitor p27, are responsible for the recently described MEN4 syndrome, that clinically overlaps MEN1 but differs from it for milder clinical features and an older patient’s age at onset, but it can explain just 1–3.5% of MEN1 genetic-negative patients. A proportion of MEN1 phenocopies could be explained by mutations of other genes predisposing to neuroendocrine tumors, or by deletions, or mutations in the regulatory regions of *MEN1* gene [4, 6–9]. Moreover, aberrant epigenetic regulation of MEN1 gene may be another potential etiology underlying mutation-negative MEN1 patients [1]. Last but not least, a reasonable explanation for at least part of MEN1 phenocopies could be simply found in the sporadic co-occurrence of two MEN1-related major manifestations, quite frequent in the general populations, namely PHPT and PitNET [10]. In fact, PHPT prevalence is relatively high accounting for 0.2% and 1.3% of the population, with an incidence rate ranging from 16 per 100,000 to 120 per 100,000 person-years globally [11]. The occurrence of PitNET has also increased over the recent years with an estimated prevalence of 76 to 116 cases per 100,000 individuals, and incidence of 3.9 and 7.4 cases per 100,000 per year in the general population [12].

Besides some of the aforementioned hypotheses, so far the evidence in the literature regarding the development of MEN1 phenocopies is still limited, and there is increasing debate about whether these patients and their relatives should undergo screening and the best methods for their monitoring.

The acknowledgment of phenocopies’ risks for developing MEN1-related tumors, as well as their possible relapse or metastatic spread, together with the unknown necessity to conduct a familial screening, often leads to an excess of morphological exams for patients, that influence their quality of life and expose them to radiological risks [13, 14]. The aim of our retrospective multicenter study is to analyse in depth the clinical presentation of MEN1 phenocopies in order to highlight their peculiarities and differences with respect to the genetic syndrome.

## Materials and methods

This is an observational, retrospective, multicenter study including adult patients clinically affected with MEN1 syndrome with negative mutational status of *MEN1* gene, i.e. MEN1 phenocopies, who referred to 10 Italian NET-referral centers during the last five years. The study was conducted in the setting of Neuroendocrine Tumors Innovation in Knowledge and Education (NIKE) project. The inclusion criteria were: (1) MEN1-like phenotype characterized by at least 2 typical MEN1 manifestations including primary hyperparathyroidism (PHPT), pancreatic neuroendocrine tumor (PanNET) or pituitary adenoma (PitNET) or (2) at least one typical MEN1 manifestation diagnosed below the age of 40 years old and/or multifocal/multiglandular disease or one classical MEN1-related disease plus at least one non classical MEN1-related disease and (3) negative genetic testing for *MEN1* mutation, by next generation sequencing (NGS) and multiplex ligation-dependent probe amplification (MLPA) [4]. We included also patients with NET other than PanNET when associated with a typical MEN1 manifestation, including PHPT and/or PitNET.

Participants’ data was collected and managed anonymously by all centers using a shared database, composed of several sections. The first one was dedicated to general demographic (age and gender) and anthropometric (weight, height and BMI) information. The second section comprised family cancer history and data from genetic screening, in particular genetic testing for *MEN1* and *CKDN1B* genes. Subsequently, a section including clinical and biochemical evaluation at diagnosis and during follow-up, therapies and pathological features when available, was designed for each of the most common manifestations of MEN1 (i.e. PHPT, NET and PitNET), to obtain data concerning functional status, clinical manifestations and outcomes. NET tumor staging was defined according to AJCC/TNM 8th ed., and grading was assessed in relation to ki67 and mitotic count according to the 2022 NENs WHO classification [15, 16]. Comorbidities were recorded. In addition, each participating center was asked to report the demographic data of patients with a genetic diagnosis of MEN1 who accessed the center for the first time within the same time frame (past five years). Such information was included in a dedicated section of the database. The study was performed in accordance with the ethical standards of the Declaration of Helsinki. Before the enrollment in the study, informed consent was obtained by participants for collecting and using their data anonymously.

### Statistical analysis

Descriptive statistics were used to describe patients’ demographic and anthropometric characteristics, comorbidities and familial oncological history, as well as the biochemical, clinico-pathological features and therapies of MEN1 typical manifestations. Continuous variables were presented as median with interquartile range when non-normally distributed and as mean ± standard deviation (SD) when normally distributed. Categorical data were reported as absolute numbers and/or percentages. Moreover, differences between phenocopies and genetic MEN1 patients were evaluated by the t-test or the Mann-Whitney U test respectively for normally and non-normally distributed continuous variables and the chi - square test or Fisher’s exact test for categorical variables. An α-error of 0.05 was established.

## Results

Sixty-five patients (44 females, 68%; 21 males, 32%; χ2 = 8.14, *p* = 0.0043) met the inclusion criteria for the study (Table 1). They were aged 18–81 years, with a median of 63 years and a mean of 59.9 *±* 11.6 years. Fifty-nine/65 (90.7%) were included because of two MEN1 manifestations, one patient for young diagnosis of PHPT, one for multi-gland PHPT disease, one for multifocal PanNET, three for one classical MEN1-related disease plus at least 1 non classical MEN1-related disease. The median age at the first disease manifestation was 54 years (aged 18–79 years, mean value 53.9 years).

**Fig. 1:**
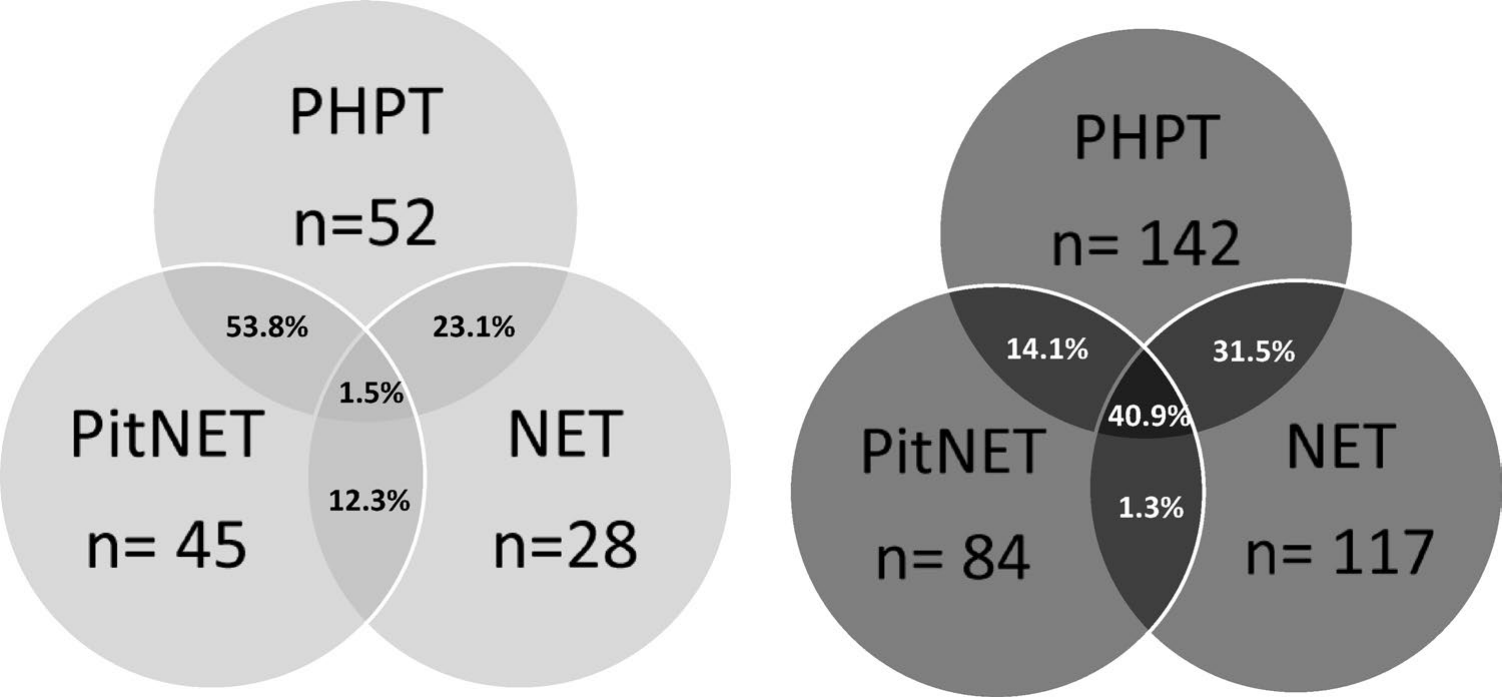
MEN1-associated disease in population negative for *MEN-1* genetic test (gray) and in population positive for *MEN-1* genetic test (black). *PTHP: primary hyperparathyroidism; PitNET: pituitary neuroendocrine tumor; NET: neuroendocrine tumor. The number below each circle is related to affected patients*

The most frequent MEN1-related disease was PHPT (52/65, 80%), followed by PitNET (45/65, 69%) and NET (28/65, 43.1%). The most common association of MEN1-related disorders was the combination PHPT with PitNET (35/65; 53.8%), followed by association between PHPT and NET (15/65; 23.1%), and PitNET with NET (8/65; 12.3%). Only one patient had all three classical diseases (1.5%) (Fig. 1). Interestingly, the first diagnosed disease was PitNET in 29 patients (44.6%) followed by PHPT in 26 patients (40%) and NET (10/65, 15.4%). Moreover, 19 patients developed one or more non classical MEN1 tumors, such as adrenal adenoma (13/65, 20%), meningiomas (7/65, 10.7%), and seminoma (1/65, 1.5%).

None of these patients had a family history of MEN1 (i.e. family members with a genetic diagnosis of MEN1). However, when considering conditions that may be associated with the MEN1 phenotype in a first-degree relative, 7/65 patients (10.7%) had positive findings: 3 patients had a first-degree relative with pitNET (1 father, 1 sister, and 1 a son), 3 patients had a first-degree relative (2 father, and 1 sister) diagnosed with NET, and 1 patient had a sister with PHPT. Overall, 33/65 (50.8%) had a personal and/or family history of cancer (breast, lung, kidney, not-NET pancreatic or gastro-enteric tumors, skin and thyroid cancers).

Over the same five-year period, 175 patients were diagnosed with genetically confirmed MEN1 at the Italian participant centers (101 females, 57.71%; 74 males, 42.29%; χ2 = 4.16, *p* = 0.041). Overall, among the 240 patients assessed for possible MEN1 syndrome, 27% were ultimately classified as phenocopies. The prevalence of phenocopies varied substantially across centers, ranging from 7% to 50%.

Regarding genetic MEN1 patients in comparison with phenocopies, mean age at diagnosis was significantly different (43.18 ± 19.66 years; *p* < 0.0001), while sex distribution was not (*p* = 0.16; χ2 = 1.97), but females were over-represented in both groups. To date, 26 patients (14.9%) have no clinical manifestations of the syndrome and were identified through genetic screening as relatives of the affected patients. Among symptomatic patients, the most common association of MEN1-related disorders was the combination of PHPT with both PitNET and NET (61/149, 40.9%), followed by the association between PHPT and NET (47/149, 31.5%), PHPT with PitNET (21/149, 14.1%) and PitNET with NET (2/149, 1.3%). Moreover, 13/149 (8.7%) had only PHPT, 2/149 (1.3%) had multiple NETs, 5/149 only NET (3.3%). Disease associations significantly differered from phenocopies (*p* < 0.001; Fig. 1). The proportion of patients presenting with the full triad of MEN1-associated tumors (primary PHPT, NETs, and PitNETs) was 40.9% among genetically confirmed MEN1 cases, compared to only 1.5% among phenocopies. Among patients exhibiting two of the three major MEN1-associated tumors, the combination of PHPT and NETs was significantly more common in genetically confirmed MEN1 patients (31.5% vs. 23.1%), whereas the combination of PHPT and PitNET was more frequently observed in phenocopies (14.1% vs. 53.8%). This distribution reflects the differing prevalence of sporadic forms of these tumors in the general population.

Among these 65 patients, 43 underwent genetic screening for MEN4 (*CDKN1B*) and tested negative, confirming that MEN4 might account only for a very small rate of MEN1 phenocopies. The main features of these *MEN1* negative/*CDKN1B* negative subgroup is reported in Table 1, and did not differ from those of all the *MEN1* negative patients. Further genetic tests were performed in a minority of patients (*n* = 15/65). Four patients with both PHPT and PitNET tested negative for *AIP* (aryl hydrocarbon receptor interacting protein) mutations, and 11 patients with PHPT and PitNET or GEP-NET (gastroenteropancreatic NET) tested negative for *CDC73* (parafibromin gene) mutations.

**Table 1:** Main demographic, anthropometric and clinical features characteristics of our phenocopies

Typical manifestations		MEN1 negative phenocopies (*n* = 65)			Both MEN1 and CKDN1B negative phenocopies (*n* =43)		
		PTHP	PitNET	NET	PTHP	PitNET	NET
**Number of patients/total number of phenocopies**		52/65 (80%)	45/65 (69%)	28/65 (43.1%)	35/43 (81%)	29/43 (67%)	21/43 (48.9%)
**Median age at diagnosis** (years old)		59.9 (range 18–79)	52.2 (range 18–77)	56 (range 18–79)	60.45 (range 18–79)	51.51 (range 28–77)	62 (range 30–79)
**F: M** (%)		38:14 (73%:27%)	33:12 (73%:27%)	17:11 (60.7%:39.3%)	29:6 (83%:17%)	23:6 (79%:21%)	12:9 (57.1%:42.9%)
**Median BMI** (kg/m^2^)		26.64 (range 20–45.2.2)	26.17 (range 20.8–45.2.8.2)	27.16 (range 20.00–34.73.00.73)	26.27 (range 20–45.2.2)	26.73 (range 20.8–45.2.8.2)	27.2 (20–32.81.81)
**BMI** Patients number (%)	Normal (18.5–24.9 kg/m^2^)	27/52 (52%)	23/45 (51%)	10/28 (37.7%)	19/35 (54%)	16/29 (55%)	7/21 (33.3%)
	Overweight (25–29.9 kg/m^2^)	18/52 (35%)	15/45 (27%)	8/28 (28.6%)	9/35 (26%)	9/29 (31%)	6/21 (28.6%)
	Obese (≥30 kg/m^2^)	7/52 (13%)	7/45 (15%)	6/28 (21.4%)	7/35 (20%)	7/29 (24%)	5/21 (23.8%)
**Smoking habit** Patients number (%)	Non-smokers	43/52 (83%)	36/45 (80%)	15 (53.6%)	30/35 (86%)	22/29 (76%)	9/21 (42.9%)
	Ex-smokers	4/52 (7.7%)	4/45 (8.8%)	4 (14.3%)	1/35 (2.8%)	2/29 (6.9%)	3/21 (14.3%)
	Smokers	5/52 (9.6%)	5/45 (11%)	3 (10.7%)	4/35 (11%)	5/29 (17%)	3/21 (14.3%)
**Comorbidites** Patients number (%)	DM	15/52 (29%)	14/45 (31%)	7 (25%)	10/35 (28.6%)	9/29 (31%)	7/21 (33.3%)
	CV disease	21/52 (40%)	18/45 (40%)	16 (57.1%)	14/35 (40%)	12/29 (41%)	13 (61.9%)

***Abbrevations***: PTHP: primary hyperparathyroidism; PitNET: pituitary neuroendocrine tumor; NET: neuroendocrine tumor; F: female; M: male; BMI: body mass index; DM: diabetes mellitus; CV: cardiovascular

In the following sections, we describe the clinical features of the MEN1-related diseases, mainly focusing on patients who were negative to both *MEN1* and *CKDN1B* tests. When comparing these *MEN-1*/*CKDN1B* negative patients with the whole group of *MEN1*-negative patients, no significant differences emerged in the main clinical features of the related disorders.

### Primary hyperparathyroidism

The main clinical and biochemical features of PHPT in our series of phenocopies are reported in Table 2. Among the 43 menin/*CDKN1B* negative patients, 35 patients received a diagnosis of PHPT (81.4%; 29 F and 6 M). Medium age at diagnosis was 60.4 years (range 18–79 years), comparable to medium age at diagnosis in the general population and with two decades of delay in comparison to MEN1 patients [17]. All patients had sufficient vitamin D levels (>30 ng/mL) at biochemical evaluation, and most of them had just mild hypercalcemia with calcium corrected for albumin below 12 mg/dL (17/35, 48.6%) (Table 2). Noteworthy, multi-gland disease was reported only in one patient.

The main clinical manifestations of the disease were osteoporosis (15/35, 42.9% of patients) and nephrolithiasis (12/35, 34%). Anyway, no patient had severe osteoporosis (T-score on lumbar column or on femur’s neck <- 4 at MOC-DEXA analysis), and a few patients experienced bone fractures. No one suffered from nephrocalcinosis and just 2 patients had chronic kidney disease stage 2 following KDIGO definition, while the others were stage 1 [18].

**Table 2:** Biochemical and clinical characteristics of PTHP

PTHP *n*. of patients with PHPT/total number of phenocopies		*MEN1* negative patients (*n*=52/65)	Both *MEN1* and *CKDN1B* negative patients (*n*=35/43)
**Sex**		F = 38; M =14	F= 29; M= 6
**Medium PTH level at diagnosis** *(normal values: 15–65 pg/mL)*		154 pg/mL (range 73–669)	155 pg/mL (range 73–490,7)
**Serum calcium levels** (mg/dL)	Normal	13/52 (25%)	4/35 (11%)
	Mildly elevated	20/52 (38%)	17/35 (48,6%)
	Elevated	19/52 (36%)	14/35 (40%)
**Medium urinary calcium** (mg/24 h)		254 (range 100–510)	278.59 (range 100–510)
**Clinical manifestations**	**Osteoporosis** (F: M ratio)	25/52 (48%) (19:6)	15/35 (42.9%) (13:2)
	**Vertebral fractures** (F: M ratio)	4/52 (7.7%) (4:0)	3/35 (8.6%) (3:0)
	**Nephrolithiasis** (F: M ratio)	21/52 (40%) (15:6)	12/35 (34%) (9:3)

***Abbrevations***: PTHP: primary hyperparathyroidism; F: female; M: male; n.:number

* Normal values: 8.5–10.5 mg/dl; mild elevate hypercalcemia < 12 mg/dl; elevated: *≥* 12 mg/dl

### PitNET

In Table 3 there are summarized the main clinical and biochemical features of PitNET in *MEN1* negative/*CDKN1B* negative subjects, in comparison with all the *MEN1* negative patients. Twenty-nine out of 43 patients who tested negative for *MEN1*/*CDKN1B*, received a diagnosis of PitNET (67%, 23 females and 6 males). Medium age at diagnosis was 51.5 years (range 18–77 years), about one decade before PHPT. In fact, PitNET was often the first MEN1-related manifestation in our cohort (20/45, 44%). Nineteen PitNETs were functioning (65,5%; F: M = 16:3): 9 growth hormone (GH)-secreting (47%; F: M = 9:0), 8 prolactin (PRL)-secreting (42%; F: M = 6:2), 2 adrenocorticotropic hormone (ACTH)-secreting PitNETs (10,5%; F: M = 1:1). One-third of patients (2 PRLomas, 4 GHomas and the 2 ACTHomas) received surgery, while 2/3 received fist-line medical therapies, accordingly to current guidelines. More in detail, among PRL-secreting, 2 patients received surgery (25%) and one needed to be also treated with gamma-knife radiotherapy, the other one needed medical treatment with cabergoline for relapse; the other 6 patients showed good clinical response to cabergoline used as first-line treatment. Among GH-secreting, 4 were treated with surgery (44.4%), but 3 needed first generation somatostatin analogue (SSA) for PitNET relapse (75% of surgical treated) and 2 also the administration of pegvisomant; the other 5 were treated with SSA as first-line therapy and one patient needed to undergo pasireotide because of resistance to first generation SSA (20%). All ACTH-secreting PitNETs received surgery, but one needed osilodrostat to control Cushing-syndrome’s relapse.

**Fig. 2:**
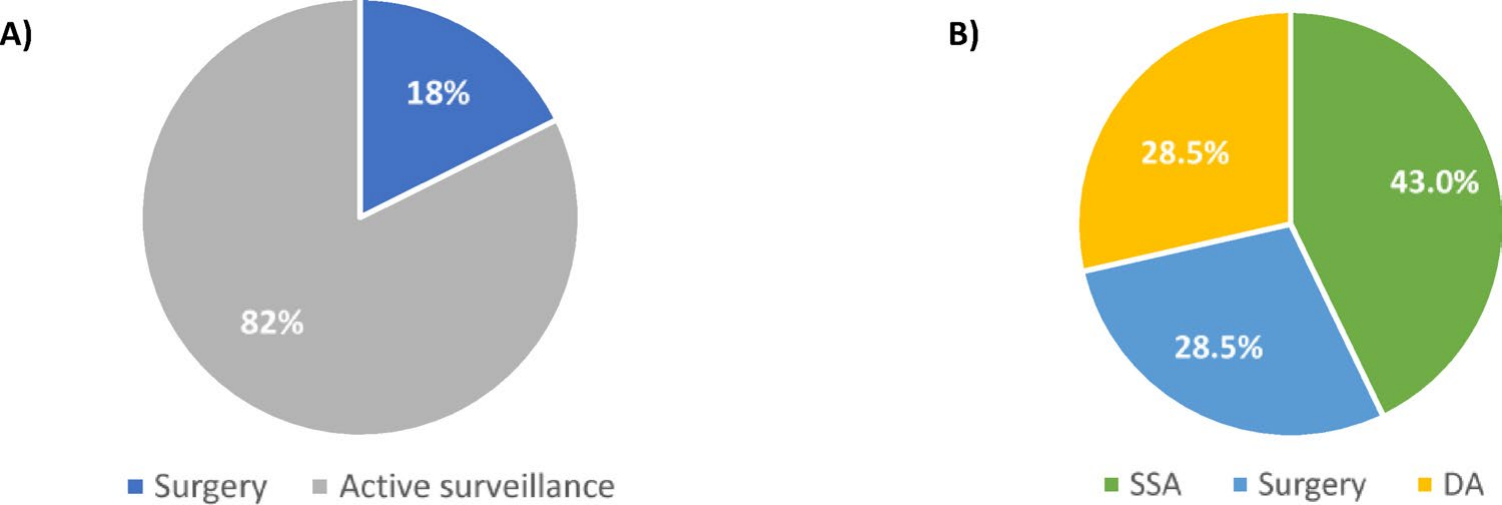
First-line therapy for **A)** non-functioning PitNETs (n=17/45) and **B)** functioning PitNETs (n=28/45) in MEN1 phenocopies. *Abbreviations: PitNET: pituitary neuroendocrine tumor; SSA: somatostatin analogue; DA: dopamine agonist*

The non-functioning PitNETs were 10/29 (34.5%; F: M = 7:3); three patients underwent surgery (30%), while the others received just active surveillance. The therapeutic approaches for both functioning and non-functioning PitNETs in all MEN1 phenocopies are illustrated in Fig. 2.

When comparing these *MEN1*/*CKDN1B* negative patients with the whole group of *MEN1*-negative patients, a higher prevalence of GH-secreting compared to PRL-secreting PitNETs emerged (16/28, 35.6% vs. 10/28, 22%), highlighting the trend of this population to receive more often diagnosis of acromegaly rather than of PRLoma. Moreover, there was one patient who had GH and PRL-secreting PitNET (1/28, 6%; F).

**Table 3:** Clinical characteristics of PitNET

PitNET *n*. of patients with PitNET/total number of phenocopies		*MEN1* negative phenocopies (*n*=45/65)	Both MEN1 and CKDN1B negative phenocopies (*n*= 29/43)
**Sex**		F = 33; M =12	F= 23; M= 6
**Functioning**	Total (%); F: M	28/45 (62%); 20:8	19/29 (65.5%); 16:3
	GH (%); F: M	16/28 (35.6%); 10:6	9/19 (47%); 9:0
	PRL (%); F: M	10/28 (22%); 8:2	8/19 (42%); 6:2
	ACTH (%); F: M	2/28 (4.4%); 1:1	2/19 (10.5%); 1:1
	GH+PRL (%); F: M	1/28 (2.2%); 1:0	0/19 (0%)
**Functioning** **First line therapy**	Surgery	8/28 (28.5%)	8/19 (42%)
	SSA	12/28 (43%)	5/19 (26%)
	Dopamine agonist	8/28 (28.5%)	6/19 (31.6%)
**Non-functioning**	Total; F:M	17/45 (38%); 13:4	10/29 (34.5%); 7:3
**Non-functioning: First line therapy**	Surgery	3/17 (18%)	3/10 (30%)
	Active surveillance	14/17 (82%)	7/10 (70%)

***Abbrevations***: PitNET: pituitary NET; NET: neuroendocrine tumor; F: female; M: male; GH: growth hormone; PRL: prolactin; ACTH: adrenocorticotropic hormone; SSA: somatostatin analogue; n.:number

### NET

**Fig. 3:**
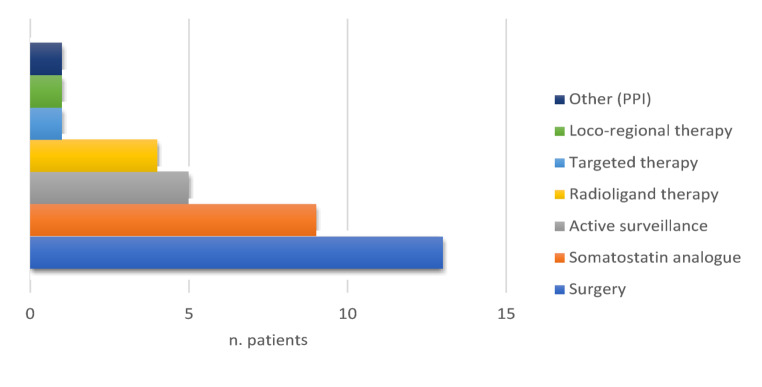
Distribution of therapeutic approaches for NETs in MEN1 phenocopy patients (*n* = 28/65)

Twenty-one (48.9%) patients among the 43 individuals with negative genetic screening for both *MEN1* and *CKDN1B* developed NETs. Information about their characteristics, surgical and pharmacological treatment is summarized in Table 4; Fig. 3.

**Table 4:** Clinico-pathological characteristics of NET in our study populations

NET *n*. of patients with NET/total number of phenocopies		MEN1 negative patients (28/65)	Both MEN1 and CKDN1B negative patients (21/43)
**Sex**		F = 17; M =11	F= 12; M= 9
**Primary tumor site** n. of cases (%)	Pancreas	17/28 (60.7%)	14/21 (66.7%)
	Intestinal tract	8/28 (28.6%)	7/21 (33.3%)
	Gastric	1/28 (3.6%)	1/21 (4.8%)
	Bronchopulmonary	4/28 (14.3%)	Not included
**Grading of the primary tumor site** n. of cases (%)	G1	11/28 (39.3%)	9/21 (42.9%)
	G2	5/28 (17.9%)	3/21 (14.3%)
	Unknown	12/28 (42.9%)	12/21 (57.1%)
**Functioning** n. of cases (%)	Carcinoid syndrome	2/8 (25.0%)	1/7 (14.3%)
	Insulinoma	3/8 (37.5%)	3/7 (42.9%)
	Hypergastrinemia	1/8 (12.5%)	1/7 (14.3%)
	GH ectopic secretion	1/8 (12.5%)	1/7 (14.3%)
	ACTH ectopic secretion	1/8 (12.5%%)	1/7 (14.3%)
**Non-functioning** n. of cases (%)		20/28 (71.4%)	13/21 (61.9%)
**Association with typical MEN1 features** n. of cases (%)	PHPT	15/28 (53.6%)	12/21 (57.1%)
	PitNET	11/28 (39.3%)	8 (38.1%)
**Therapy**	Active surveillance	5/28 (17.9%)	4/21 (19.0%)
	Surgery	13/28 (46.4%)	9/21 (42.9%)
	SSA	9/28 (32.1%)	5/21 (23.8%)
	RLT	4/28 (14.3%)	2/21 (9.5%)
	Targeted therapy	1/28 (3.6%)	0/21 (0%)
	Loco-regional therapy	1/28 (3.6%)	1/21 (4.8%)
	Other pharmacological therapy (PPI)	1/28 (3.6%)	1/21 (4.8%)

***Abbrevations***: NET: neuroendocrine tumor; PHPT: primary hyperparathyroidism; PitNET: pituitary neuroendocrine tumor; SSA: somatostatin analogue; RLT: radioligand therapy; PPI: proton-pump inhibitor; GH: growth hormone; ACTH: adreno corticotropic hormone

The most commonly used treatments included surgery and somatostatin analogues, followed by active surveillance and radioligand therapy. Less frequent approaches encompassed targeted therapy, loco-regional procedures, and proton pump inhibitors (PPIs).

In *MEN1*/*CDKN1B*-negative subgroup, women and men with NET accounted respectively for 12 (57.1%) and 9 (42.9%) (F: M = 1.3:1). The median age at diagnosis in all patients was 62 years (range 30–79), and specifically, in women it was 55 years (range 37–79), whereas in men 66 years (range 30–79). About the primary NET site, pancreas was the most frequent in both women (9/12, 75.0%) and men (5/9, 55.6%), accounting overall for 14 cases (14/21, 66.7%), of which only 1 was characterized by multiple neoplastic foci. Seven (*n* = 7, 33.3%) patients developed NET of the intestinal tract: 3 in the duodenum, 1 in the ileum, 1 in the cecum margin, 1 in the ascending colon and 1 in the appendix. Afterward, 1 subject presented gastric NET. In 2 (9.5%) subjects, different NET types arose either synchronously or metachronously: one patient developed pancreatic, gastric and bronchopulmonary NETs, while the other one developed pancreatic NET and paraganglioma of the left carotid bifurcation. In terms of grading, available data of 12 patients showed that all NETs were G1/G2 tumors. Most cases (13, or 61.9%) were non-functioning NETs, whereas 7 cases (5 women and 2 men) were associated with clinical syndromes, the most common being hypoglycemia due to insulinoma (Table 4). The majority of phenocopies diagnosed with NETs underwent surgery (up to 46.4%), followed by SSA therapy (up to 32.1%) and active surveillance (up to 19.0%). A minority, instead, required second or third-line medical treatment such as RLT (up to 14.3%) or targeted therapy (up to 3.6%).

### Non classical MEN1-related features

Among the 43 patients with genetic screening negative for both *MEN1* and *CKDN1B*, there were 9 cases (20.9%) of adrenal nodules. When considering the whole MEN1-negative population (*n* = 65), 5 (7.7%) cases of meningiomas and 1 case of seminoma were identified, in addition to a total of 13 (20%) cases of adrenal nodules (Table 5).

About the typical MEN1 features, among all patients with adrenal nodules, 7 presented also PTHP, 4 GEP-NET, and 6 PitNET. In this cohort, there was a combination of PTHP and PitNET in 4 cases, PTHP and GEP-NET in 3 cases, while GEP-NET and PitNET in 1 case.

**Table 5:** Main atypical MEN1-related manifestation in both study populations

Adrenal nodules		MEN1 negative phenocopies	MEN1 and CKDN1B negative phenocopies
**N.patients/total phenocopies**		13/65 (20.0%)	9/43 (20.9%)
**Gender F: M**		10:3 (76.9%:23.1%)	8:1 (88.9%:11.1%)
**Median age** (years old)		57 (42–92)	65 (50–92)
**Median BMI** (n. patients)	Normal (18.5–24.9 kg/m^2^)	3/13 (23.1%)	3/9 (33.3%)
	Overweight (25–29.9 kg/m^2^)	4/13 (30.8%)	2/9 (22.2%)
	Obese (≥30 kg/m2)	5/13 (38.5%)	4/9 (44.4%)
**Functioning**	Yes	5/13 (38.5%)*	4/9 (44.4%)*
	No	6/13 (46.2%)	5/9 (55.6%)
	CV disease	7/13 (61.5%)	4/9 (44.4%)
**Association with MEN1 typical manifestations**	PTHP	10/13 (76.9%)	7/9 (77.8%)
	PitNET	7/13 (53.8%)	6/9 (66.7%)
	NET	7/13 (53.8%)	4/9 (44.4%)
	PTHP + PitNET	5/13 (38.5%)	0/9 (0%)
	PTHP + NET	4/13 (30.8%)	4/9 (44.4%)
	NET + PitNET	1/13 (7.7%)	1/9 (11.1%)

***Abbrevations***: F: female; M: male; BMI: body mass index; DM: diabetes mellitus; CV: cardiovascular; PTHP: primary hyperparathyroidism; PitNET: pituitary neuroendocrine tumor; NET: neuroendocrine tumor; NA: not available

* Of the 9 cases of adrenal nodules in *MEN1*- and *CKDN1B*-negative patients, 4 (44.4%) were functioning (2 cases associated with hypercortisolism, 2 pheochromocytomas). About the 13 cases observed in the whole MEN1-negative cohort, only 5 were functioning, including the aforementioned 4 cases and 1 case of Conn disease

### Oncological familial history in phenocopies with negative genetic screening for *MEN1* and *CKDN1B*

None of the 43 patients with negative genetic screening for both *MEN1* and *CDKN1B* presented a family history of MEN1. Among all 43 subjects, 16 (37.2%) had oncological family history (PitNET, NET, thyroid, breast, lung, colon, gastric, prostate, pancreatic, laryngeal cancer). In particular, 3 patients had a first-degree relative with PitNET, 2 of whom had non-functioning adenoma (father and 1 a son) and 1 not known if functioning (1 sister). One patient had a family history of small bowel NET (1 sister), 4 patients of breast cancer (1 mother, 1 a daughter, 2 unknown), 3 patients of lung cancer (1 both grandparents, 1 father, 1 an uncle), 1 patient of colon cancer (father), 1 patient of gastric cancer (unknown), 1 patient of prostate cancer (unknown), 1 patient of laryngeal cancer (mother), 1 patient of thyroid cancer (1 sister), 1 patient of parathyroid adenoma (unknown). One (*n* = 1) patient had family history of pancreatic cancer (both father and a paternal relative), while 1 of pancreatic neoplasia not further specified.

## Discussion

MEN1 phenocopies are not uncommon, with an estimated prevalence ranging from 10 to 30% of patients with clinical MEN1, and therefore they represent an important clinical challenge [4]. Due to the limited epidemiological data, the precise frequency of phenocopies, particularly when comparing sporadic cases to familial ones, remains undetermined. Nevertheless, negative genetic tests are observed more commonly in sporadic than in familial cases [10, 19].

In the present study we analyze the clinical, familial, and genetic profiles of a cohort of 65 Italian patients with a MEN1 phenocopy; 70% of these individuals were also tested and found out to be negative for *CDKN1B* mutation, confirming the rarity of MEN4 syndrome. Over the same five-year period, 175 patients were diagnosed with genetically confirmed MEN1. Overall, among the 240 patients assessed for possible MEN1 syndrome, 27% were ultimately classified as phenocopies. The prevalence of phenocopies varied substantially across centers, ranging from 7% to 50%. Notably, an inverse relationship was observed between the number of genetically confirmed MEN1 cases and the proportion of phenocopies in individual centers: the higher the number of genetically confirmed MEN1 patients diagnosed at a center, the lower the proportion of phenocopies. This variability likely reflects differences in diagnostic experience and case selection among centers, suggesting that referral bias and the availability of genetic testing may influence the proportion of phenocopies observed.

Overall, the clinical characteristics of genetically confirmed MEN1 patients in this cohort are consistent with those reported in a large recently published Italian multicenter study, supporting the robustness and representativeness of our findings. Thus, we sought to compare our findings with available data on Italian patients with genetically confirmed MEN1, to further elucidate potential differences in phenotype, clinical course and prognosis between mutation-negative and mutation-positive individuals [20].

Similar to the MEN1 Italian subjects, our phenocopies were predominantly women (F: M = 2:1; *p* = 0.0043) but with an overall median age at diagnosis of 63 years (range 18–82), that is approximately 2 decades later than in mutation-positive MEN1 patients, aligning more closely with the general population [20, 21]. Even in our genetic-MEN1 group women were significantly over-rappresented, and age at diagnosis was significantly lower than phenocopies (*p* < 0.0001).

In our cohort of phenocopies, the most common clinical combination was PHPT and PitNET (53.8%), followed by PHPT and GEP-NET (23.1%), with only one patient presenting all three classical MEN1-associated tumors. Conversely, the complete MEN1 tumor triad was observed in 40.9% of genetically confirmed cases, underscoring the diagnostic value of tumor clustering. Among patients with two manifestations, the combination of PHPT and NET was significantly more indicative of a genetic MEN1 diagnosis, while PHPT and PitNET were more frequent in phenocopies. This pattern likely reflects the higher baseline prevalence of sporadic pituitary tumors in the general population and highlights the limited specificity of certain tumor combinations in the absence of genetic confirmation. Similarly, in the Italian MEN1 database by Giusti et al., which included 475 MEN1 patients (15.8% *MEN1* mutation-negative), patients developed predominantly an association of PHPT and NET (114 cases, 24%), followed by the association of all three manifestations (92 cases, 19.4%) and finally PitNET and NET (8 cases, 1.7%) [20]. This difference is not surprising since PHPT and PitNET are relatively common in the general population. Notably, in our phenocopies, PitNET was the first diagnosed tumor in 45% of cases, compared to PHPT in 40%. This contrasts with genetic MEN1 patients, where PHPT is both the most frequent and earliest manifestation. In fact, the Italian MEN1 study identified PHPT as the most frequent manifestation (93%, *n* = 405) as well as the first diagnosed one (67%). NETs (53%, 230), instead, were the second most frequent feature, followed by PitNETs (41%, 178) [20].

In order to highlight potential similarities and differences, we compared the clinical manifestations of each MEN1-related disease, and overall phenotypic profile of our patient cohort with those reported in the Italian MEN1 dataset [20]. PHPT was the most prevalent manifestation (80%), often presenting with osteoporosis, albeit with lower-than-expected severity for age and disease stage. This rate (about 40%) was between the one reported for patients aged 26–50 years (25%) in the Florentine MEN1 database and the one observed in those over 51 years (57.1%) [22]. Moreover, bone involvement appeared less severe than expected, with lower rates of vertebral fractures and absence of markedly reduced bone density (T-score < −4). Additionally, nephrolithiasis and chronic kidney disease were less frequent, suggesting a milder PHPT phenotype compared to genetic MEN1. In 71.2% of cases, PHPT was diagnosed due to clinical symptoms, unlike the typically asymptomatic presentation in MEN1 patients. The rarity of multiglandular involvement further supports the potential benefit of conservative surgery in these patients.

PitNETs in phenocopies were mostly functioning, with GH-secreting tumors being predominant, differing from the prolactinoma predominance seen in MEN1. A substantial proportion (36.8%) required second-line therapy, indicating a potentially aggressive course similar to that observed in genetic MEN1 [23]. Conversely, non-functioning PitNETs were largely managed with active surveillance, reflecting a generally indolent behavior unusual for PitNET in MEN1 patients [23, 24].

As regards NET patients, NETs showed a female predominance and were diagnosed at an older age compared to MEN1 patients. They were mainly non-functioning and commonly associated with PHPT. When functioning, insulinomas were more frequent in phenocopies, unlike the gastrinoma predominance in MEN1. The pancreas was the most common primary site, with rare multifocal disease. Treatment strategies, including surgery (46.4%), somatostatin analogues (32.1%), and active surveillance (19.0%), closely mirrored those used in MEN1 patients, and the comparable need for second-line treatments suggests a similarly challenging clinical behavior [20].

Our data suggest that an older age at diagnosis, the absence of lack of multifocal or multiglandular lesions, and the more favorable course, at least in PHPT, represent the main distinctive features of phenocopies in comparison with *MEN1* mutation-positive patients. This is in line with previous data from the literature.

Kövesdi et al. identified the occurrence of NETs, especially at age < 30 years, as the most significant predictor of *MEN1* mutation positivity among all MEN1-related clinical manifestations. Conversely, mutation-negative patients were more likely to develop PHPT either alone or combined with PitNETs, a trend also reported by Klein et al. in 288 apparently unrelated probands [19, 25]. Real-life clinical data from the Dutch MEN Study Group (DMSG) further underscore the more favorable course of mutation-negative cases: these patients developed two out of the three primary MEN1 manifestations later in life than the MEN1 counterparts, and demonstrated a life-expectancy comparable to the general population [26]. Moreover, most of them (76.7%) presented co-occurrence of PHPT and PitNET, without developing GEP-NETs whose metastatic progression remains the leading cause of mortality in MEN1 patients [10, 27].

While confirming previous data in the literature, the most original contribution of our study lies in the identification of an increased individual and familial susceptibility to oncological diseases. Noteworthy, about 12% (7/65) of our phenocopies had at least one first-degree relative diagnosed with MEN1-associated tumors, and 50% (33/65) of them had a personal and/or family history of other cancer types, including breast, lung, kidney, non-NET GEP, skin and thyroid cancers, suggesting an increased risk for developing endocrine and non-endocrine tumors in this cohort. This finding prompts concerns about the most appropriate clinical-diagnostic management and follow-up protocols for these patients.

Our analysis, however, presents several limitations including the retrospective design of the study, the inherent potential selection bias and the small sample size. An additional drawback lies in the fact that the information concerning the genetic testing for *CDKN1B* as well as for *AIP* in patients harbouring PitNETs, was available only for a subset of subjects. Furthermore, a prospective study involving a larger cohort of patients, with data obtained from a single central laboratory, could be useful to minimize possible pre- and post-analytical variations and selection bias.

Currently, the early diagnosis and management of MEN1 phenocopies still represent a relevant clinical challenge also due to the absence of standardized guidelines. A recent ENETS MEN1 task force questionnaire study involving the Centers of Excellence (CoEs) addressed the issue of surveillance for MEN1 genotype-negative patients. For these individuals, a more limited screening and surveillance approach was overall suggested, depending also on the patient’s clinical features. Specifically, most CoEs favored either a screening strategy similar to that used for mutation-positive patients (68% of CoEs) or a personalized approach based on the patient’s clinical features (25% of CoEs). Nearly all (92%) would suggest periodic screening in phenocopies with combined duodeno-pancreatic NET with either PHPT or PitNET, while only 25% supported screening in cases with both PHPT and PitNET. A cut-off age of < 50 years was advised by the majority of CoEs (64%) for initiating surveillance. Regarding first-degree relatives of phenocopy patients, 42% of CoEs supported periodic screening, whereas 21% would advise other approaches such as education or initial screening with individualized follow-up depending on the screening outcome and patient’s clinical profile [28]. More recently, Faggiano et al. identified reliable phenotypic criteria, including young age (< 40 years), multifocality, multiple MEN1-associated manifestations (PHPH and/or pitNET), and endocrine syndromes, suggestive of MEN1 syndrome in patients with NET as first manifestation and a negative family history [29].

## Conclusions

MEN1 phenocopies are not rare. In our cohort, the three major MEN1-related manifestations were more frequent in women and were generally diagnosed in the seventh decade of life, that is approximately 20 years later than in *MEN1* mutation-positive patients. PHPT was the most common disease, but PitNETs represented the most frequent initial manifestation, having been diagnosed slightly less than 1 decade earlier than PHPT in phenocopies. NETs were mainly non-functioning, G1-G2 PanNET, and when functioning, insulinomas were the prevalent subtype, unlike MEN1 patients. The behaviour of PitNETs and NETs in phenocopies, as supported by treatment data, was comparable to MEN1 genetic patients, and further underlines the necessity of early diagnosis.

The occurrence of MEN1-associated tumors in first-degree relatives (11.6%) and a notable family history of non-MEN1 cancers suggests the potential utility of expanded genetic testing and structured screening protocols for these patients. Considering the high prevalence of PHPT and the earlier diagnosis of PitNET compared to PHPT in our study population, screening of PTH levels and calcium-phosphorus metabolism should be proposed at the initial recognition of PitNET. However, the lack of specific guidelines continues to hinder standardized care. There remains considerable debate regarding the appropriateness of screening these patients and the optimal strategies for their monitoring. Further studies are warranted to better understand the biological underpinnings of MEN1 phenocopies, which is essential for timely diagnosis and appropriate management of mutation-negative individuals.

## Data Availability

The datasets generated during the current study are available from the corresponding author on reasonable request.
